# Methods for Measuring and Identifying Sounds in the Intensive Care Unit

**DOI:** 10.3389/fmed.2022.836203

**Published:** 2022-06-06

**Authors:** Aileen C. Naef, Samuel E. J. Knobel, Nicole Ruettgers, Marie-Madlen Jeitziner, Martin grosse Holtforth, Bjoern Zante, Joerg C. Schefold, Tobias Nef, Stephan M. Gerber

**Affiliations:** ^1^Gerontechnology and Rehabilitation Group, ARTORG Center for Biomedical Engineering Research, University of Bern, Bern, Switzerland; ^2^Department of Intensive Care Medicine, Inselspital, Bern University Hospital, University of Bern, Bern, Switzerland; ^3^Institute of Nursing Science (INS), Department of Public Health (DPH), Faculty of Medicine, University of Basel, Basel, Switzerland; ^4^Department of Clinical Psychology and Psychotherapy, University of Bern, Bern, Switzerland; ^5^Psychosomatic Medicine, Department of Neurology, Inselspital, Bern University Hospital, University of Bern, Bern, Switzerland; ^6^Department of Neurology, Inselspital, Bern University Hospital, University of Bern, Bern, Switzerland

**Keywords:** intensive care unit, noise, sound level meters, hospital, decibels, sound pressure levels, sound sources

## Abstract

**Background:**

Despite many studies in the field examining excessive noise in the intensive care unit, this issue remains an ongoing problem. A limiting factor in the progress of the field is the inability to draw conclusions across studies due to the different and poorly reported approaches used. Therefore, the first goal is to present a method for the general measurement of sound pressure levels and sound sources, with precise details and reasoning, such that future studies can use these procedures as a guideline. The two procedures used in the general method will outline how to record sound pressure levels and sound sources, using sound level meters and observers, respectively. The second goal is to present the data collected using the applied method to show the feasibility of the general method and provide results for future reference.

**Methods:**

The general method proposes the use of two different procedures for measuring sound pressure levels and sound sources in the intensive care unit. The applied method uses the general method to collect data recorded over 24-h, examining two beds in a four-bed room, via four sound level meters and four observers each working one at a time.

**Results:**

The interrater reliability of the different categories was found to have an estimate of >0.75 representing good and excellent estimates, for 19 and 16 of the 24 categories, for the two beds examined. The equivalent sound pressure levels (L_Aeq_) for the day, evening, and night shift, as an average of the sound level meters in the patient room, were 54.12, 53.37, and 49.05 dBA. In the 24-h measurement period, talking and human generated sounds occurred for a total of 495 (39.29% of the time) and 470 min (37.30% of the time), at the two beds of interest, respectively.

**Conclusion:**

A general method was described detailing two independent procedures for measuring sound pressure levels and sound sources in the ICU. In a continuous data recording over 24 h, the feasibility of the proposed general method was confirmed. Moreover, good and excellent interrater reliability was achieved in most categories, making them suitable for future studies.

## Introduction

Patients admitted to the intensive care unit (ICU) are constantly exposed to high sound pressure levels due to the complex nature of their treatment, involving numerous medical devices, alarms, and staff involved. Moreover, in previous research the already high sound pressure levels in the ICU have been found to be increasing by 0.38 dBA per year during the day, and by 0.42 dBA per year at night (1). The average daytime sound pressure levels have increased from 57 dBA in 1960 to 72 dBA in 2005, and night-time levels have risen from 42 dBA to 60 dBA during the same period. Consequently, sound pressure levels in the ICU continue to exceed the recommended 45 dBA during the day, and 30 dBA at night, set forth by the World Health Organization (1–8).

One of the most difficult aspects of addressing the excessive sound pressure levels in the ICU is understanding what the precise causes of the high sound pressure levels are. Literature has presented evidence suggesting that in the ICU setting, staff-generated sounds are the greatest contributor to the total sound pressure levels recorded (2, 9), particularly in close proximity to the bed (10). Other major sources of sound in the ICU are attributed to activities such as hand washing, opening packages, storage drawers, telephones, and pagers (2, 4, 11). This is in line with a study by Vreman et al. (6) which found that overall alarms only contributed to a minor proportion of overall sound pressure levels in the ICU, however, other studies suggest a more significant role of monitor alarms (2, 12, 13). Understanding the true sources of sound is of importance due to its role in patient and staff health. In patients, changes in cardiovascular performance (14), sleep disruptions (15), and adverse clinical outcomes (16) have been linked to sound pressure levels >50 dBA. Patient outcomes can also be affected by alarm fatigue and burnout experienced by ICU professional repeatedly exposed to high sound pressure levels (12, 17).

Despite the existing literature on the topic, progress in addressing the known problems in this field is slow. One reason for this is the difficulty drawing comparisons between studies addressing this topic (11, 18). The lack of a defined procedure, methods, and parameters, for measuring and identifying sound within the ICU setting has resulted in numerous papers on the topic (9, 11, 18). For example, there are differences in the number, type, and location of the devices used. Some studies may use one or two recording devices placed near the patient head (19), while others may place them closer to the foot of the bed (20), or even in the hallway (21). Alternately, there are studies which use six or more devices (2, 21). Recording duration also varies between studies, with some recording continuously (19), while others record only intervals (21, 22). The devices used for the analysis also vary, with some using microphones (10, 23) or sound level meters (19, 24), while another may use previously recorded sounds (9). Differences also exist for observers identifying sound sources, with some studies using a single observer (3) and other using multiple observers (4). The checklists used for identifying or grouping sound sources is also unique to each paper and often difficult to replicate due to a lack of details. The checklists may be made up of more generalized groups (2, 9), while others may use more detailed lists or include additional precisions per group (3, 4, 9, 25). As such, it is relevant to the field to tackle this lack of consistency so that progress can be made in reducing high sound pressure levels, an issue that concerns hospitals worldwide (1, 2, 4, 9, 26).

For this reason, the current paper aims to standardize the assessment of sound levels and sound sources in the ICU, outlining a generalizable method with repeatable procedures and clear parameters. By providing a detailed description of the proposed steps, as well as the reasoning behind the choices, the goal is to provide a baseline that can be used for future investigations into the sound pressure levels and sound sources in the ICU. To show the feasibility of the general method, and to act as a reference, data from a 24-h recording period at two beds, in a four-bed patient room, collected using the proposed procedures, is presented. It is expected that by using the proposed procedures as a guideline, future studies will not only be able to successfully record sound pressure levels and sound sources in the ICU environment for single- and multi-day experiments, but also be comparable among each other.

## Materials and Methods

**Figure 1:**
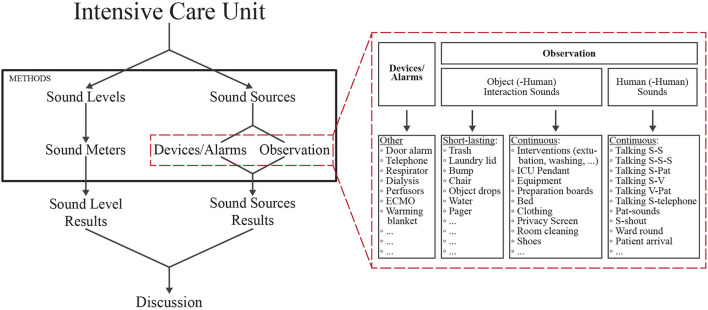
Schematic outlining the two parallel methods for examining sound in the ICU setting. Left: The sound pressure levels in the ICU can be determined objectively using sound level meters. Right (red dashed box): details showing how the sound sources in the ICU can be determined via device alarms or via observers. These two components of sound in the ICU can be combined to create an overall picture of sound in the ICU. Staff (S); patient (Pat); visitor (V); extracorporeal membrane oxygenation (ECMO); intensive care unit (ICU). Full list of abbreviations, sound sources and descriptions can be found in the Supplementary Table 1.

The general method is composed of two procedures that can be conducted simultaneously but independently (Figure 1). One procedure outlines how sound pressure levels can be measured using sound level meters, while the second procedure outlines how to identify sound sources using observers. These two procedures will be described in more detail below.

### General Method

#### Sound Pressure Levels Procedure

##### Sound Equipment

Sound pressure levels are recorded using sound level meters. To be consistent with the literature, it is recommended that the decibel range of the device is appropriate for the sounds of interest (for example 30–90 dB). At least one sound level meter should be used per bed, plus one additional sound level meter outside of the patient room to capture sound in the surrounding environment. This means that if measuring a four-bed patient room, to examine all four beds four devices would be required, one above each bed, plus one for the outside the patient room, such as in the hallway. For recording using the sound level meters, there are a number of settings possible. It is recommended that the A-weighted filter is selected as it discriminates against low and high frequencies, comparable to the response of the human ear, and is standard weighting for indoor measurements (6, 27). Additionally, a fast time weighting is also recommended due to its higher resolution and similarities to the integration time of the human ear. The equivalent continuous sound level represents a stead sound pressure level around which the actual noise level fluctuates over a given time. When using A-weighted measurements, the equivalent sound level is denoted as L_Aeq_ (Supplementary Equation 1) (28). Similarly, the maximum, fast, A-weighted sound level measured (L_AFmax_) represents the maximum sound pressure level reached during a defined recording period, using an A-frequency weighting and a fast time weighting.

For higher data quality, and to be able to record over longer periods, it is important that the devices are placed in an easily accessible place near a power-source. Certain devices need a continuous power source and have limited space for saving the data, therefore, data must be downloaded from the devices regularly, without interrupting the daily work of the ICU staff. Additional considerations pertaining to the individual device is whether the device can be disinfected, what the appropriate size is for the setting, and how it can be placed in or attached to the desired location. To ensure accurate sound pressure level recordings it is important that the sound level meters are placed in such a manner that they are not quickly noticed in an attempt to limit influencing the hospital staff. If the sound level meters are too easily noticed, this may create a bias in that sound pressure levels decrease (2, 4).

#### Medical Equipment

Within the ICU setting there are various stationary and movable devices which may be in a patient room at any given time. Many of these devices provide the possibility to extract the alarm information retrospectively, directly from the device itself, thereby easing the task of human-observers tracking the sound sources. For example, depending on the device, it may be possible to collect the occurrence of alarms concerning hemodynamics, dialysis, oxygenation, and perfusions following the observation period. Other patient-related alarms such as heart rate, blood pressure, blood saturation, and respiration rate, could also potentially be collected via the vital sign monitoring system. Miscellaneous alarms, such as doorbells or emergency reanimation alarms, might also be logged. Therefore, a query into the ability of retrieving such information from devices should be performed prior to beginning any observation period.

#### Sound Source Procedure

##### Checklist Development

To create a sound source checklist a dual bottom-up approach, based on the literature and clinical observations, was used. A baseline list of sound sources in the ICU was generated using previous work on the topic (2–4, 25). Next, an explorative approach was chosen to determine what types of sound sources exist in the patient room, both from a patient and staff perspective. This was done by having a member of the study team shadow a nurse working in the ICU for several h. Following this observation period, the findings were compared to and merged with those noted in the literature.

As the number of sound sources increased, it became unrealistic to be able to identify and note everything in parallel. Furthermore, sounds had to be differentiated into continuous and short-lasting sounds. To reduce complexity, observation periods were separated into five-min intervals and sound sources were grouped as either human (-human) or object (-human) interactions. Subsequently, the categorization was then tested and further adapted as new sounds were added and sounds were shifted from one classification to the other.

To further ease the challenge of noting all sound sources individually, clusters were created. For example, clusters such as equipment, preparation board, medical pendant, and interventions were created. A preliminary list of clusters was then assembled (Supplementary Table 1), to which new items could be added during the observation period.

#### Parameters and Scoring

**Figure 2:**
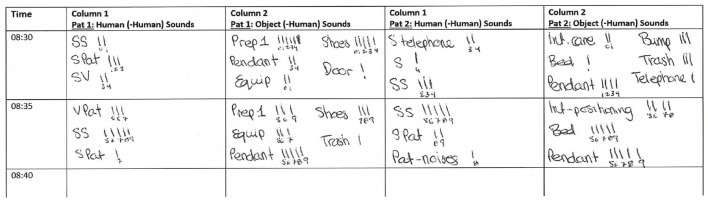
Example observer sheet showing how two beds would be documented. The two columns on the left represent bed 1, and the two columns on the right represent bed 2. For each bed, the left column is where human (-human) sounds such as talking should be listed. For each bed, the right column is where object (-human) sounds should be noted such as sounds coming from the equipment, medical pendant, or shoes. Staff (S); staff-staff (SS); staff-patient (SPat); staff-visitor (SV); visitor-patient (VPat); preparation board one (Prep1); equipment (Equip); patient-noises (Pat-noises); intervention (Int). Full list of abbreviations, sound sources, and descriptions can be found in the Supplementary Table 1.

The classification of sounds as continuous and short-lasting during the development of the checklist was important for defining how the groupings should be scored. Continuous sounds are those considered to last anywhere from more than a few sec to a min and should be scored as a duration. If a continuous sound occurs within a 5-min interval, the category of the sound (e.g., pendant, intervention, etc.) is noted, and a stroke is written down to indicate that it occurred in a single min. If the sound occurs again in the same min (e.g., from 8:30–8:31) no new stroke is added. If, however, the sound occurs again in a different min (e.g., from 8:31-8:32) another stroke is added (Figure 2). If a sound is categorized as continuous, a maximum of five strokes can be reached, representing that a sound occurred at most for 5 min. To make it easier for the observer and to avoid mistakes, the min in which a sound occurs can be noted as a small number under each stroke (Figure 2). This way, when the same sound is captured, it is quickly recognizable whether a new stroke must be made, or whether it still counts toward the already existing stroke. For continuous sounds, a score for each sound source is then achieved by adding up the total number of min the sound was heard which represents the maximum length of time it could have been heard.

On the other hand, if a short-lasting sound is heard, it should be scored as a frequency, and every occurrence should be noted by adding a stroke to the observation sheet (Figure 2). Here the number of strokes is not limited to five. Short-lasting sounds are those that cannot be continuous. For example, if a door closes or an object is dropped the sound cannot last more than a few sec. For short-lasting sounds, the total number of occurrences can be determined by adding up all the strokes.

#### Observer Shifts

When considering what type of observer shifts to use, the first step would be to decide how long the measurement period should last, and how many people are available to act as observers. The advantage of having multiple observers is the ability to continuously record sound sources over longer time periods and potentially even over multiple days. It also ensures the ability to maintain concentration levels during times of peak activity and avoid alarm and noise fatigue (12, 13). For this reason, daytime observers should alternate in their shifts. To help further decrease fatigue, observers should also be allowed a 10-min break every h, during which they can leave the room. Changes in observers should occur during these breaks as it allows the study team a chance to debrief, clarifying certain questions, or provide updates which may help the subsequent observer. If possible, observer changes should also not occur during staff shift changes to avoid missing data for these known periods of high sound pressure levels.

The length of shifts presented in the literature ranges from 10 min (3) to 3 h (26), with some also alternating observers (5). While it could be argued that shorter shifts may be better for maintaining concentration, there are other advantages to longer observation shifts. For example, the more time spent in the patient room, the more familiar the observer becomes with what is occurring in the room. As there is often a lot happening simultaneously it can be overwhelming when an observation shift begins, as the observer must first familiarize themselves with the current situation in the room. While some studies cite the involvement of nurses and doctors (4), we propose that the observers can be anyone familiar with the hospital setting.

Based on these factors, using four observers, the following observation shifts are proposed as an example, with breaks always occurring from:20 to:30 every h:

Observer 1: 6:30–8:20, 11:30–14:20, 17:30–20:20

Observer 2: 8:30–11:20, 14:30–17:20

Observer 3: 20:30–1:20

Observer 4: 1:30–6:20

It should be noted that it is recommended that objective measurements using sound level meters overlap temporally with the data collected by human observers pertaining to sound sources. This way it is possible to support any claims based on subjective measurements with objective recordings.

#### Interrater Reliability

Based on guidelines outlined by Koo and Li (29) the intraclass correlation coefficient (ICC) estimates and their 95% confidence intervals should be calculated based on a mean-rating (*k* = number of raters), absolute-agreement, two-way-mixed-effects model. Scoring suggestions provided by Koo and Li (29) should also be followed to interpret the results. Estimates above 0.90 are considered excellent and estimates between 0.75 and 0.90 are considered good. Those between 0.50 and 0.75 are moderate, and those below 0.50 are poor. It should be noted that when the variance in the samples is low, in this case the ICC is likely to be low as well, or cannot even be calculated (30).

### Applied Method

#### Sound Pressure Level and Source Procedure

To show the feasibility of the proposed methods, and provide data for future comparisons, a reference recording was carried out. The reference recording was conducted for 24-h, with four observers and four sound level meters, looking at two beds in a four bed ICU room.

#### Interrater Reliability

Prior to conducting the reference recording, the interrater reliability of the four observers was tested. A total of 6 h were recorded: three during the day shift (8:00–11:00) to represent a busier observation period, and three during the evening shift (20:00–23:00) to represent a calmer observation period. During each three-h period, each of the four observers was compared to the other three observers, for a total of 30 min overlap per observer pair. ICC calculations were subsequently completed using the psych package in R version 4.0.4 (R Core) (31) and the two-way mixed-effects model and scoring described in section Interrater Reliability were used. For ease of computation, and to increase the number of data points for analysis, the individual items scored were grouped into categories. The detailed list of the categories can be found in Supplementary Table 2.

#### Setting

The preliminary scoring for interrater reliability, and the reference 24-h recording, was performed in the Department of Intensive Care Medicine in a mixed medical-surgical ICU at the University Hospital Bern, Switzerland. The corresponding department of Intensive Care is the sole provider for adult critically ill patients in the tertiary care academic center (University Hospital of Bern, Inselspital). Assessments were performed in a 16-bed subunit. The methods presented in this paper were approved by the local ethics committee via waiver as no identifiable patient data was collected (KEK 2020-01294).

**Figure 3:**
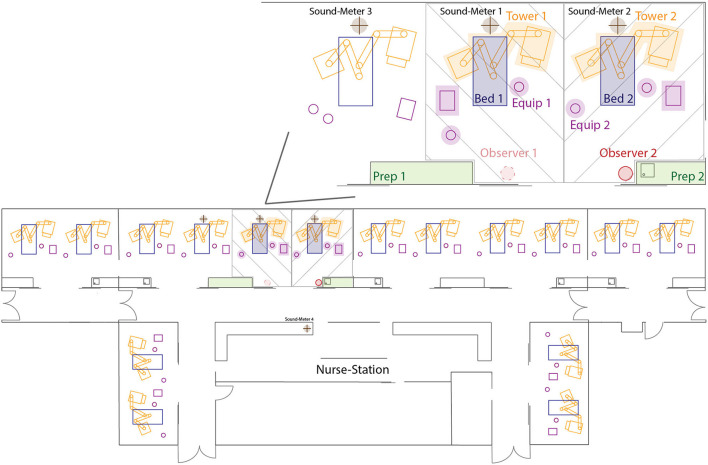
Schematic display of the ICU ward on which the measurements took place. In the upper right, the two beds examined for the 24-h reference recording and inter-rater reliability are displayed in more detail. Observer 1 chair was only present while collecting data for the interrater reliability test. Green zones represent the preparation board, the pink items represent free-standing equipment in the room, orange zones represent the medical pendant on either side of the bed, the placement of the patient bed is depicted in dark blue, and the crossed brown circles represent the location of the sound level meters above the beds and at the nursing station. Shaded areas represent the two bed places examined for the reference recording and interrater reliability. Full list of abbreviations can be found in the Supplementary Table 1.

The mixed medical-surgical ICU at the University Hospital Bern has three ICU wards with similar structural designs. The ward chosen for these assessments has 16 beds spread across double-bed and four-bed rooms, with a central nursing station outside the patient rooms (Figure 3). The four-bed rooms are bracketed by a two-bed room on one side, and a four-bed room on the opposite side (Figure 3). These rooms are connected via sliding doors which are generally left open to create a sense of unity between the spaces. There are also two large double sliding doors at either end of the ward, at the foot end of the beds, leading into the main corridor. The central nursing station is located across the corridor from these large sliding doors, permitting ICU staff at the station to maintain a view of the patient beds (Figure 3). To obtain a representative sound measure, two neighboring beds in the four-bed room, on the side closest to the adjoining four-bed room, were selected for reference observations (shaded area Figure 3). These positions were selected as they are located near the middle of the ward, thereby, providing a representative measure of sound pressure levels throughout the entire ward. To provide naturalistic conditions for the 24-h reference recording, beds could be occupied or unoccupied over the recording period. For this reason, no patient was present in bed 2 for around four and a half h, approximately between 10:45–15:15.

For every two beds in a room, there are two preparation boards inside the room, one with a sink and one without (Figure 3). These preparation boards are mainly used to prepare medications, and to store and unpack material. At each bed, there are also two -medical pendants, one on each side of the bed (Figure 3). Nurses use these pendants to administer medications and during patient care, as well as to access patient data and update electronic patient charts via an integrated patient data management system. In the hallway directly outside the four-bed room there are additional, mobile, preparation boards and equipment that can be used in the hallway or brought directly into the patient room. The described setup may not be equivalent across all ICUs, but the goal was to propose a method that is as independent as possible. Nevertheless, there may be certain differences in the individual setting and researchers should make the appropriate changes to the proposed methods based on their own setup.

#### Sound Equipment

For the reference recording presented in this paper, a total of four sound level meters were used. Three devices were class II personal sound dosimeters (Extech-SL400, Extech Instruments, USA) and one was a class I sound level meter (PCE-430, PCE Germany GmbH, Germany). Generally speaking, class I sound level meters are referred to as ‘precision' grade, whereas class II meters are referred to as ‘general purpose' (27, 32). This is related to the fact that class I devices have a wider frequency range and are considered as being more accurate than class II devices due to their narrower tolerances at the higher frequencies (27). However, for most applications these differences are not noticeable. All devices were calibrated by the manufacturer and were used with a sample rate of 1 Hz.

Each of the three class II sound level meters were placed above one of three beds (Figure 3). The sound level meters were attached directly above the head-end of the patient bed, at a height of approximately 2.5 meters above the ground and 0.4 meters from the ceiling. Due to the moveable nature of the bed and medical pendants on either side of the bed, it was decided not to attach the sound level meter directly to these structures. Placing the sound level meters above the bed also meant that the positioning was consistent over the duration of the 24-h recording as it was not attached to a single bed and would not be moved if a patient was admitted or discharged, or if the space was cleaned.

Another consideration is that in multi-bed rooms, it is possible that beds close to the wall may be quieter as they only have a neighboring patient on one side. As can be seen in Figure 3, the setup looked at two beds in the same room, one toward a wall, and one toward the middle of the room, with the two directly beside one another. The third, class II, device was placed above the neighboring bed to act as a control so it could be determined if loud sounds were coming from that side of the room. The reasoning was that if sound level meter 3 measured higher decibels than sound level meters 1 and 2 in Figure 3, it could be concluded that something producing high sound pressure levels was occurring on the left side of the room.

The class I device was placed in the hallway at the nurses' station located directly outside the patient room of interest (Figure 3). Since it was expected that sounds from outside the patient room would cause higher peaks than inside the room, the class I device was placed in the hallway as it had a slightly larger decibel range than the class II devices. The device was placed in such a manner that it was minimally noticeable to anyone walking past the nurses' station.

#### Data Collection

Four observers conducted the 24-h continuous reference recording, each working alone. The observer sat to the right of the door as depicted in Figure 3 (see observer 2). For the reference recording, the observer shifts described in section Observer Shifts were used, with the end time extended by30 min. This was done so that the data could be analyzed by shift, with a start time of 7:00, which corresponded to the start of the morning shift in this particular hospital. While all sounds were noted individually and scored as described in section Parameters and Scoring, further groups, as done for the ICC calculations, were made for ease of the final analysis. A detailed list of groupings can be found in Supplementary Table 2. All sounds that were logged either directly in the equipment itself, or in a database, were not additionally noted by the observer in the room.

To verify that the presence of the observer did not influence the sound pressure levels measured, follow-up recordings were conducted for an additional 24-h, 8, 15, and 22 days after the original recording day. Follow-up measurements were done with sound level meters only, with no observer present in the room. The follow-up measurements also ensured that the day measured was representative of the norm, and not by chance much louder or quieter.

## Results

### Interrater Reliability

ICC estimates >0.90 (excellent) were achieved for 12 of the 24 categories which were heard during the day (Table 1). ICC estimates greater than 0.90 were achieved for eight of the 24 categories which were heard during the evening (Table 1). ICC estimates between 0.75 and 0.90 (good) were achieved for seven of the 24 categories during the day and eight of the 24 categories during the evening. ICC estimates between 0.50 and 0.75 (moderate) were achieved for two of the 24 categories during the day and for one of the 24 categories during the evening. ICC estimates below 0.50 (poor) were achieved for one of the 24 categories for both day and evening. No estimate could be computed for one of the 24 categories during the day and for six of the 24 categories during the evening due to lack of variance due to random effect. There was one category which did not occur during the day but did occur at evening, so no estimate was given for the day. Full results from the ICC can be found in Table 1.

**Table 1:** Full ICC estimates obtained using a two-way mixed-effects model during the day and evening observation periods.

	**Description**	**Day**			**Evening**		
		**Estimate**	**F(*p*)**	**95% Interval**	**Estimate**	**F(*p*)**	**95% Interval**
Human (-Human) Sounds	Staff < 3 people talking (out of ward round)	0.93	13 (*p* < 0.001	0.89–0.95	0.95	20 (*p < 0.001*)	0.93–0.97
	Patient talking	0.95	19 (*p* < 0.001)	0.92–0.96	0.98	54 (*p < 0.001*)	0.97–0.99
	Staff ≥ 3 people talking (out of ward round)	0.94	17 (*p* < 0.001)	0.91–0.96	0.27	1.4 (*0.094*)	0.70–0.86
	Patient Sounds	0.96	26 (*p* < 0.001)	0.93–0.97	0.77	4.4 (*p < 0.001*)	0.66–0.85
	Staff sounds	0.66	3 (*p* < 0.001)	0.50–0.77	–	–	–
	Staff during ward round ≥ 3 people talking	0.99	90 (*p* < 0.001)	0.98–0.99	0.80	4.9 (*p < 0.001*)	0.70–0.86
	Staff during ward round < 3 people talking	0.99	190 (*p* < 0.001)	0.99–1.00	0.80	4.9 (*p < 0.001*)	0.70–0.86
Object (-Human) Interaction Sounds	Oxygen	0.99	120 (*p* < 0.001)	0.99–0.99	0.99	122 (*p < 0.001*)	0.99–0.99
	Pendant	0.90	10 (*p* < 0.001)	0.85–0.93	0.89	9.1 (*p < 0.001*)	0.84–0.93
	Preparation board	0.96	26 (*p* < 0.001)	0.94–0.97	0.95	21 (*p < 0.001*)	0.93–0.97
	Clothing Accessories	0.84	6.2 (*p* < 0.001)	0.76–0.89	0.50	2 (*0.0015*)	0.27–0.66
	Free standing equipment	0.82	5.7 (*p* < 0.001)	0.74–0.88	0.90	10 (*p < 0.001*)	0.85–0.93
	Diagnostic	0.98	40 (*p* < 0.001)	0.96–0.98	1	5.1e+14 (*0*)	1.00–1.00
	Bed–related	0.87	7.6 (*p* < 0.001)	0.81–0.91	1	5.1e+14 (*0*)	1.00–1.00
	Unknown intervention	–	–	–	–	–	–
	Continuous maintenance	1	4.9e+14 (*0*)	1.00–1.00	–	–	–
	Admission and discharge	0.96	25 (*p* < 0.001)	0.94–0.97	–	–	–
	Activity of daily living: non–mobilization	0.98	46 (*p* < 0.001)	0.97–0.99	0.96	25 (*p < 0.001*)	0.94–0.97
	Activity of daily living: mobilization	0.89	9.4 (*p* < 0.001)	0.84–0.93	0.97	39 (*p < 0.001*)	0.96–0.98
	Privacy screens	0.79	4.9 (*p* < s)	0.69–0.86	–	–	–
	Nursing	N/A	N/A	N/A	–	–	–
	Short–lasting activities	0.45	1.9 (*0.0046*)	0.20–0.63	0.82	5.6 (*p < 0.001*)	0.73–0.88
	Short–lasting maintenance	0.78	4.5 (*6.8e−10*)	0.67–0.85	0.84	6.4 (*1e−13*)	0.76–0.89
	Ringing	0.73	3.7 (*4.9e−8*)	0.60–0.82	0.80	4.9 (*7.9e−11*)	0.70–0.86

*Detailed explanations of the groups can be found in Supplementary Table 2. Estimate represents the estimated correlation value; F(p) represents the F statistic and its associated p-value; 95% Interval represents the 95% confidence interval of the estimate; N/A (not applicable) indicates the sound was heard during one, not both, of the observation periods, while ‘-' indicates that there was not enough variance in the data to compute the ICC*.

### Applied Sound Pressure Level and Source Results

#### Sound Pressure Levels

**Figure 4:**
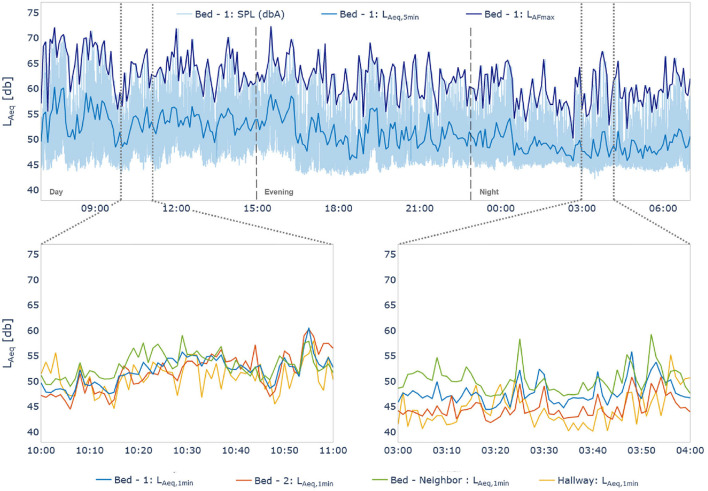
(Top) 24-h overview of sound pressure levels measured by the class II device positioned over bed 1 (Figure 3). Dark blue shows the L_FAmax_, and the light blue trend shows the L_Aeq_ calculated over 5-min. The shaded area represents the raw sound pressure levels (SPL). (Bottom) Sub-figures show 1-minL_Aeq_ values for all devices (one class I, three class II) illustrating the different levels of alignment between the devices over two 1-h snapshots. Vertical dashed lines (15:00 and 23:00) indicate shift changes.

Analysis of the 24-h reference recording found elevated sound pressure levels across the three shifts (Figure 4). Using the sound pressure levels recorded by the class I device placed at the nurses' station the L_Aeq_ values, corresponding to the average A-weighted sound energy received over time, were calculated to be 52.55, 51.06, and 49.00 dBA during the day, evening, and night shifts, respectively. Recordings from the three class II devices placed in the patient room resulted, as an average of the three devices, in L_Aeq_ levels of 54.12, 53.37, and 49.05 dBA during the day, evening, and night shifts, respectively. The maximum sound pressure levels measured with an A-frequency weighting and fast-time weighting (L_AFmax_), were 76.50, 85.59, and 79.47 dBA for the class I device, during the day, evening, and night shifts respectively. For the same shifts, the average L_AFmax_ of the class II devices were 71.20, 72.20, and 66.87 dBA. For full results per shift see Table 2.

**Table 2:** Overall time of sound source occurrence in min over the 24-h observation period, and percent occurrence per 8-h shift.

	**Description**	**Bed 1**				**Bed 2**			
		**Overall**	**Day**	**Evening**	**Night**	**Overall**	**Day**	**Evening**	**Night**
Human (-Human) Sounds	Staff < 3 people talking (out of ward round)	299 min	21.88%	35.66%	13.81%	284 min	40.94%	24.58%	1.90%
	Patient talking	141 min	-	11.33%	11.43%	116 min	12.47%	11.33%	3.81%
	Staff ≥ 3 people talking (out of ward round)	67 min	6.59%	9.40%	-	91 min	18.59%	2.89%	-
Object (-Human) Interaction Sounds	Oxygen	279 min	-	25.90%	30.95%	272 min	-	34.22%	30.95%
	Pendant	193 min	14.82%	19.52%	11.67%	212 min	22.12%	24.58%	3.81%
	Preparation board	290 min	21.41%	28.67%	19.05%	101 min	14.59%	8.91%	1.19%
	Monitor Alarms	245 min	10.8%	12.3%	29.8%	177 min	9.4%	15.0%	12.3%
	Clothing accessories	126 min	13.41%	7.95%	8.57%	126 min	17.41%	9.64%	2.86%

*The top three human (-human) sounds and top five object (-human) interaction sounds, as determined by the total sum between the two beds, are shown here. Results for each category can be found in Supplementary Table 4*.

Results from the follow-up recordings, during the 24-h recording and eight, fifteen-, and twenty-2-days post-recording, found L_Aeq_ levels of 50.87, 51.55, 51.68, and 52.05 dBA, from the device in the hallway at the nurses' station. The average decibel levels of the three devices above the beds in the patient room were 51.83, 51.97, 52.99, and 50.57 dBA, for the same time periods. The L_AFmax_ values on the initial recording day and follow-ups were 79.85, 78.82, 76.88, and 76.72 dBA for the device in the hallway, and 70.26, 71.02, 73.62, and 68.84 dBA for the devices above the beds. For full results per shift see Supplementary Table 3.

#### Sound Sources

Overall, during the 24-h reference recording, for bed 1 (Figure 3) talking and human generated sounds occurred for 495 min, making up 39.29% of the time (Table 2). For bed 2 (Figure 3), talking and human generated sounds occurred for 470 min, making up 37.30 % of the time (Table 2). For bed 1, other sound sources which were responsible for making up the highest percentage of sounds were the preparation board (290 min, 23.02%), oxygen related patient interventions (279 min, 22.14%), monitor alarms (245 min, 17.6%), the pendant (193 min, 15,32%), sounds coming from staff clothing like shoes and accessories on clothing (126 min, 10.00 %), and free-standing equipment in the room (89 min, 7.06 %) (Table 2). In the area around bed 2, the sound sources responsible for making up the highest percentage of sounds were oxygen related patient interventions (272 min, 21.59%), the pendant (212 min, 16.83%), monitor alarms (177 min, 12.3%), free standing equipment in the room (132 min, 10.48%), diagnostic interventions like measuring temperature or conducting an x-ray (128 min, 10.16%), sounds coming from staff clothing like shoes and accessories on clothing (126 min, 10.00%), and the preparation board (101 min, 8.02%) (Table 2).

## Discussion

In this paper we address two aspects related to measuring sound pressure levels and sound sources in the ICU. First, we fill a gap in the literature on the topic of sound pressure level measurements in the ICU. Currently, studies on the topic are inconsistent in terms of the sound level meters used, placement of recording devices, and parameters for the recordings, making it difficult to reproduce the work (3, 5, 26). Second, studies about the sound sources in the ICU environment lack comparability due to poor study documentation and differences in which parameters were observed, scoring criteria, and the number of observers (9, 11, 18). To address these shortcomings, we have demonstrated a feasible, and reliable method for multi-day measurements which is adaptable to individual needs in a constantly changing environment.

### Interrater Reliability

Overall, the interrater reliability estimates for these sounds were largely found to be high, with the majority of the estimates falling into the strong and almost perfect categories. Measuring the interrater reliability is important to ensure the accuracy and feasibility of the proposed methods for collecting data regarding the ICU sound sources. Another important reason for examining the interrater reliability of the observers is to determine the extent of bias introduced by having changing observers (4, 5). Having a high interrater reliability decreases the probability of bias being introduced by using multiple observers. This is important as it allows for a longer period of time to be observed as the study is not limited to a single observer (3). In addition to not being limited to a single observer, by confirming a high interrater reliability prior to the start of the study, replicability can be enhanced.

While the ICC estimates measured in the study are already quite high, there are still some categories, such as clothing accessories and garbage and laundry bins, that could be improved. However, slight differences in scoring are to be expected, for example, at the beginning of an intervention or due to prior knowledge of the ICU environment. An observer familiar with the intervention may recognize it sooner and score it as such, whereas an observer less familiar with such an intervention may continue scoring individual aspects of the scene before recognizing it as pertaining to an intervention. Therefore, to improve the interrater reliability, it is recommended that non-medically trained observers spend time prior to the study observing the ICU environment so that they are qualified to identify the different sound sources.

### Sound Pressure Level Measurements

The results of this study are consistent with the literature (4, 5, 9, 11). Namely, sound pressure levels exceed those recommended by health authorities, and as expected, sound pressure levels decrease in the evening and at night compared to during the day (Figure 4; Table 2). While the data presented here only represents a single day, it can be assumed that by continuously recording for longer periods, the day-to-day and weekly variations in sound pressure levels could accurately be captured and assessed. Another advantage of recording sound pressure levels continuously over days and weeks is that it could capture work shift (i.e., day, evening, night) and staff related changes in sound pressure levels. It would be interesting to confirm whether the trends seen in the different work shifts presented here occur daily and over longer-periods.

Our proposed setup and procedure for recording sound pressure levels in the ICU environment addresses current concerns about the lack of clear, long-term, measurement protocols (9, 18). Not only is the proposed method replicable, but it is also adaptable based on specific needs making it independent of any given ICU or specific patient room. This is an important consideration so that future studies on the topic are comparable, more robust, and generalizable. Moreover, the 24-h reference recording provides a reference measurement structure. Compared to previous studies, the proposed method also provides a more accurate representation of the entire sound situation by continuously measuring the sound pressure levels, not only focusing on peak sound pressure levels (3).

As can be seen in the results, there are moments during the 24-h reference recording where the three class II devices align quite well despite their physical distances, and moments where they differ quite substantially. Therefore, a limitation of this design is that there is no correct number of devices which can be recommended. For example, if looking at just the data from the left sub-figure (10:00–11:00), one might conclude that due to the similar recordings of the three devices, only one would have sufficed. However, looking at the right sub-figure (3:00–4:00) it appears that there is on average a 5 dBA difference between device 1 and device 3, thereby supporting the use of the three devices.

Future analyses will be specific to the goals of each individual investigation. However, based on the literature, we propose some basic guidelines, which can be applied to sound pressure level data to draw preliminary conclusions, regardless of the final analyses. First, we propose the calculation of the L_Aeq_ and L_AFmax_ using 5-min epochs to generate a trend representative of the data. Second, we recommend analyzing the data based on 5- min windows. Taking a window that is too large may mask relevant data, however, taking a window that is too small may make interpretation more difficult due to an increased complexity and high number of peaks which may mask the high general sound levels. Moreover, in the case that data is also collected from observers, the 5-min epochs would match those of the observer. Third, it would be relevant to examine the data based on work-shifts present in the ICU of interest. There are staff and organizational differences between work shifts which may play a role regarding sound pressure levels. Therefore, we propose examining the variables of interest such as maximum sound pressure level or average sound pressure level, taking work shifts into account.

### Sound Source Measurements

Consistent with the literature, major sound sources found using the presented method are conversations among the staff, patient interventions, monitor alarms, as well as equipment and devices in the room (2, 5, 9). While there were slight differences between the two beds considered, the absence of a patient in bed 2 for approximately four and a half h likely played a major role. Between the two beds, many of the sources generating the most sound were consistent, such as oxygen related interventions, monitor alarms, the medical pendants, free standing equipment in the room, and the preparation boards. Unfortunately, a lack of detailed descriptions and over generalized groupings in the literature make more precise comparisons to previous studies difficult.

In addition to facing problems generalizing data, previous studies examining the same question also faced several limitations. For example, studies with one, or multiple, in person observers may have generated a bias due to their presence in the study room influencing how the staff worked (2, 4). The bias here was limited in a few ways. First, the nursing team present for the observation period was informed of the study prior to its start and were also asked to continue working as normal, ignoring the study team. The staff were also asked not to interact with the observer, and informed that nothing being written down by the observer could not be link to specific individuals.

Bias was further reduced by having the observer present continuously. Even if the ICU staff adapted their behavior when the observer was first present in the room, it is unlikely that they would have been able to sustain such a change for more than a short time. This would have been especially difficult in the stressful, life-or-death situations often encountered in the ICU. This is supported by the results from the follow-up recordings, which were obtained in the absence of an observer and were in line with the values recorded when the observer was present. This supports the notion that even if a slight influence of the observers is present initially, it is likely not sufficient to generate a noticeable influence the sound pressure levels in the room.

Similarly, we propose some basic guidelines for how analyses of sound source measurements should be conducted. More specifically, we propose two basic methods for examining the data of interest, based on how it was scored. Both methods can be adapted based on the recording duration of interest, ranging from 5-min epochs to multiple days. For continuous data scored as duration, it is recommended to present the results as the total min it was noted for and calculate the percentage of the total recording time. For example, if talking is scored as occurring for 720 min (twelve h), this would correspond to 50% of the total 24-h recording time. For short-lasting sounds scored as frequency, it is recommended to calculate the number of occurrences for each category of interest. While previously presented in the literature, definitions used for the analysis often lacked (e.g., over how long the percentage was calculated). Thereby, by using the procedure described in this paper it would become easier to compare results across studies, which will be important in decreasing sound pressure levels in the ICU.

## Conclusion

Elevated sound pressure levels are an ongoing problem in the ICU, with few comparable studies on the topic. Here a clearly defined, reliable, and replicable method for both measuring sound pressure levels and sound sources in this highly complex setting, both short- and long-term, is presented. Moreover, a 24-h recording is provided for future studies to use as a reference and shows that it is feasible to conduct a recording using the proposed method.

## Data Availability Statement

The raw data supporting the conclusions of this article will be made available by the authors upon reasonable request.

## Author Contributions

AN, NR, SK, and SG designed the study, collected data, analyzed the data, and were involved in the writing, editing and reviewing of the manuscript. M-MJ, MH, BZ, JS, and TN assisted in the data collection, study design, and were involved in editing and reviewing the manuscript. All authors contributed to the article and approved the submitted version.

## Funding

Open access funding was provided by the University of Bern.

## Conflict of Interest

M-MJ, BZ, and JS report grants from Orion Pharma, Abbott Nutrition International, B. Braun Medical AG, CSEM AG, Edwards Lifesciences Services GmbH, Kenta Biotech Ltd., Maquet Critical Care AB, Omnicare Clinical Research AG, Nestle, Pierre Fabre Pharma AG, Pfizer, Bard Medica S.A., Abbott AG, Anandic Medical Systems, Pan Gas AG Healthcare, Bracco, Hamilton Medical AG, Fresenius Kabi, Getinge Group Maquet AG, Dräger AG, Teleflex Medical GmbH, Glaxo Smith Kline, Merck Sharp and Dohme AG, Eli Lilly and Company, Baxter, Astellas, Astra Zeneca, CSL Behring, Novartis, Covidien, Hemotune, Phagenesis, Philips Medical, Prolong Pharmaceuticals, and Nycomed outside the submitted work. The money received was paid into departmental funds. No personal financial gain applied. The remaining authors declare that the research was conducted in the absence of any commercial or financial relationships that could be construed as a potential conflict of interest.

## Publisher's Note

All claims expressed in this article are solely those of the authors and do not necessarily represent those of their affiliated organizations, or those of the publisher, the editors and the reviewers. Any product that may be evaluated in this article, or claim that may be made by its manufacturer, is not guaranteed or endorsed by the publisher.
