# Erratum: Methods for measuring and identifying sounds in the intensive care unit

**DOI:** 10.3389/fmed.2023.1183690

**Published:** 2023-03-21

**Authors:** Frontiers Production Office

**Affiliations:** Frontiers Media SA, Lausanne, Switzerland

**Keywords:** intensive care unit, noise, sound level meters, hospital, decibels, sound pressure levels, sound sources

An omission to the funding section of the original article was made in error. The following sentence has been added: “Open access funding was provided by the University of Bern.”

The original article has been updated.

