# Supplementary material for: Methods for Measuring and Identifying Sounds in the Intensive Care Unit
Source: Front Med (Lausanne). 2022 Jun 6;9:836203. doi: 10.3389/fmed.2022.836203 (PMC9207602; doi:10.3389/fmed.2022.836203)
Supplement: Supplementary file 1 [file Data_Sheet_1.PDF]

Supplementary Material

1 Supplementary Figures and Tables

1.1 Supplementary Equations

Supplementary Equation 1. Equation depicting the calculation of the A-weighted equivalent continuous sound level (L_{Aeq})

$$L_{Aeq,T} = 10 \log_{10} \left(\frac{1}{T} \int_{0}^{T} \left(\frac{P_A(t)}{P_0} \right)^2 \mathrm{d}t \right)$$

Where $P_A(t)$ is the acquired A-weighted sound pressure, P_0 is the reference pressure level (typically 20µPa, and T is the time period in hours representing the measurement duration.

1.2 Supplementary Tables

Supplementary Table 1. List of sound sources for bedside assessment. This list is not fixed, and can be modified and completed according to individual study settings.

<table>
<tr><td>Objective of bedside observation</td><td>Collect all observed sound sources in an intensive care unit (people talking, cleaning, object falling, intervention at the patient, ward rounds, etc.), except the ones extractable from medical devices (e.g. monitor alarms)</td></tr>
<tr><td>Patient bed/area of observation</td><td>Define area of observation (number of patient beds, area that defines patient area)</td></tr>
<tr><td>Individuals present</td><td>According to the setting, specify the individuals present as precisely as possible (e.g., individuals distinguishable via uniforms):
S = staff
Healthcare staff:
N = nurse
P = physician
Ph = physiotherapist
RT = radiological technologist
…
Non-healthcare staff:
C = cleaning staff
T = technician
…
E = unknown/extern (e.g., priest, police)
Pat = patient
V = visitor
O = observer (member of the study team)</td></tr>
<tr><td colspan="2"><h2>Part A: Human (-human interaction) sounds</h2>Sound generation due to people making verbal and non-verbal sounds, enter data into column 1 of the observation sheet (see Figure 2)

Continuous human sound:
Code with strokes (I – IIIII) for each minute in which the sound occurs.
Code sound lasting a few seconds up to one minute, occurring within a same minute (e.g., 0 min to 0 min. 59 sec.) with one stroke.
If sound occurs several times in the same minute, code with only one stroke
If sound occurs again in the next minute or continues into the next minute, code with two strokes.
There are maximum five strokes in a five-minute observation interval.
Indicate the minute of occurrence with a number below the stroke</td></tr>
</table>

Examples for interval 0 min. to 4 min. 59 sec.:
I = occurs in minute 0
0

I I I = occurs in minutes 0, 1 and 3
0 1 3

Examples for interval 5 min. to 9 min. 59 sec.:
I I = occurs in minutes 6 and 8
6 8

I I I I I = occurs in minutes 5, 6, 7, 8 and 9
5 6 7 8 9

Source	Abbreviation	Definition
A1. Verbal		
Shout	*Shout*	Call/shout from Staff over long distances, from the room into the corridor or over a patient bed
Talk	*S*<br>*SS (Change 2)*<br>*SSS (Round 8)*<br>*SSSS*<br>*5S*<br>*6S*<br>…<br>*SPh*<br>…<br><br>*SPat*<br>*SSPat*<br>*PhPat*<br>…<br><br>*VPat*<br>*VVPat*<br>*SV*<br>*SVVPat*<br>…<br><br>*SE*<br>*SO*<br>…	All conversations between people present:<br>• Specify who is/are talking (see above for abbreviations)<br>• If there is a special event known for talking, specify the event (i.e., ward round, shift change, teaching, etc.) and the number of people attending<br>• If SS are talking and a third S joins the conversation, code *SS* and additionally *SSS*, even if it is in the same minute.<br>• If Staff is talking to a Patient who can/does not answer, code *SPat.*<br><br>Examples:<br>*S* = one person from staff talks to themselves<br>*SSSS* = four Staff talk to each other (or 2x 2 persons talk separately)<br>*SS (Change 2)* = two people from Staff talk to each other during the shift change.<br>*SSS (Round 8)* = eight people from Staff are attending the ward round but only three talk<br>*SSPat* = two Staff talk with/to Patient<br>*PhPat* = Physiotherapist talks with/to Patient<br>*SVVPat* = Staff and two Visitors talk with/to Patient<br>*SE* = Staff talks with External person<br>*SO* = Staff talks to/with Observer
Talk on the telephone	*S-Phone*<br>*Pat-Phone*<br>*V-Phone*<br>…	All telephone conversations<br>• Specify who is/are talking (see above)<br>Example:<br>*S-Phone* = Staff talks on the telephone

A2. Non-verbal		
Staff/ Visitor/ Extern / Observer's cough	*S-cough*<br>*V-cough*<br>*E-cough*<br>*O-cough*	Clearly audible cough or sneeze<br>• Specify who is coughing (see above)<br>• If Patient coughs: Pat-sound (see below)<br><u>Example</u>:<br>*V-cough* = visitor coughs
Staff/ Visitor/ Extern's non-verbal sound	*S-sound*<br>*V-sound*<br>*E-sound*<br>…	All human sounds which are not from conversations or cough: laughing, clapping, drumming hands on body, deliberately clicking pen, etc.<br>• Specify who generate the sound (see above)<br><u>Example</u>:<br>*E-sound* = Extern is clapping
Patient's non-verbal sounds and cough	*Pat-sound*	All sounds coming from the patient which are not from conversations: groaning, shouting, coughing, clearly audible snoring, etc.<br>If patient talks to Staff or Visitor, code *SPat* or *VPat* (see above).

Part B: Object (-human interaction) sounds

Sound generation by object due to patient care, general activities, or maintenance, enter data into column 2 of the observation sheet (see Figure 2). Continuous (more than a few seconds) and short-lasting (less than a few seconds) sounds are distinguished.

Continuous object sound:
Code with strokes (I – IIIII) for each minute in which the sound occurs.

- Code sound lasting a few seconds up to one minute, occurring within a same minute (e.g. 0 min to 0 min. 59 sec.) with one stroke.
- If sound occurs several times in the same minute, code with only one stroke
- If sound occurs again in the next minute or continues into the next minute, code with two strokes.
- There are maximal five strokes in a five-minute observation interval.
- Indicate the minute of occurrence with a number below the stroke

<u>Examples for interval 0 min. to 4 min. 59 sec</u>.:

I = occurs in minute 0
0

I I I = occurs in minutes 0, 1 and 3
0 1 3

<u>Examples for interval 5 min. to 9 min. 59 sec.:</u>

I I = occurs in minutes 6 and 8
6 8

I I I I I = occurs in minutes 5, 6, 7, 8 and 9
5 6 7 8 9

<table>
<tr><td colspan="3">Short-lasting object sound (indicated by *):
Code with one stroke (I) for each occurrence of the sound.
If sound occurs three times within a five-minutes interval, code with three strokes.
There number of strokes in a five-minute observation interval is not limited.
<u>Examples</u>:
II = sound occurs two times within a five-minutes interval
IIIIIII = sound occurs seven times within a five-minutes interval</td></tr>
<tr><td>Source</td><td>Abbreviation</td><td>Definition</td></tr>
<tr><td colspan="3">B1. Patient care</td></tr>
<tr><td>Admission, transfer, and discharge</td><td>Admission
Discharge</td><td>Admission = Patient comes into the ICU room: initial admission and transfers (e.g., from the operating room, from the CT scan)
Discharge = Patient leaves the ICU room: transfers (e.g., to the operating room, to the CT scan) and final discharge</td></tr>
<tr><td>Interventions</td><td>Int. unknown

Int. mouth care
Int. washing
Int. feeding
Int. bedpan/urinal
Int. mobilizing
Int. physiotherapy

Int. bandage
Int. wound care
…
Int. warming blanket
Int. changing bedsheet
…

Int. blood pressure
Int. temperature
Int. ECG
Int. EEG
…
Int. X-ray
Int. sonography
…

Int. central venous catheter
Int. urinary catheter
…</td><td>All noisy medical events which are conducted with direct contact to patient (not admission, transfer, or discharge).
The intervention includes all the steps of the procedure: bring and prepare the material (corresponding to the begin of the event), perform the intervention, and put away the material (corresponding to the end of the event).
Specify the name of the intervention, if possible, otherwise code Int. unknown.
Do not code separately Prep, Equip, Pendant, Trash and Lid (see below)
If the intervention causes no sound, do not code it. Differentiate between what is heard and what is seen.

<u>Used medical abbreviation:</u>
BAL: bronchoalveolar lavage
CPR: cardiopulmonary resuscitation
ECG: electrocardiogram
ECMO: extracorporeal membrane oxygenation
EEG: electroencephalogram
IPC: intermittent pneumatic compression
RRT: renal replacement therapy (hemofiltration, hemodialysis, and peritoneal dialysis)</td></tr>
</table>

	Int. CPR<br>*Int. ECMO*<br>*Int. IPC*<br>…<br>*Int. water seal drainage*<br>*Int. oxygen*<br>*Int. suctioning*<br>*Int. intubation*<br>*Int. extubation*<br>*Int. BAL*<br>*Int. bronchoscopy*<br>…<br>*Int. RRT*<br>…	
…	…	…
B2. General activities		
Preparation board	*Prep1*<br>*Prep2*<br>*Water*<br>…	All noisy events which happen at the preparation boards: open/close drawers and cabinet doors, prepare medication, open packages, use disinfectant, open/close water tap, water running, etc.<br>Possibly define more precise sources according to individual patient room setups:<br>• *Prep1* = sounds generated at the first preparation board<br>• *Prep2* = sounds generated at the second preparation board<br>• *Water* = sounds generated by the water tap
Free standing equipment	*Equip*<br>…<br>*Supply*<br>*Computer*<br>…	All noisy events which are related to free standing equipment in the room (not at the pendant or at the preparation board): moving tables and trolleys, opening packages, carrying boxes, putting on/ taking off protective clothing, etc.<br><br>Possibly define more precise sources according to individual patient room setups:<br>• *Supply* = sound due to the supply trolley (trolley containing the material to fill up): move, close, open the supply trolley.<br>• *Computer* = sound due to the mobile computer cart (e.g., used for the ward rounds)<br>If immobile laundry/garbage bin or chair, code *Lid*, *Trash*, *Laundry,* or *Chair* (see below).<br>If related to preparing a new Patient's admission, code *Prepare (*see below).

Pendant	*Pendant*<br>…	All noisy events at the pendant, at the head-end of the patient bed, both sides: open/close drawers, prepare and administer medication, open packages, put on gloves, use disinfectant, use computer for documentation, etc.<br>Possibly define more precise sources (e.g., right or left) according to your patient room.
Bed	*Bed*	All noisy events related to the bed (not interventions, not at the pendant): raise/lower the bed, add extension, fold up/down the bed rails, move/brake the bed, straighten/put away blanket and pillow, etc.
Privacy Screens	*Divider*<br>*Door*	*Divider* = room divider is set up or rolled away<br>*Door* = door to the patient room is opened/closed
Chair*	*Chair**	Chair/stool is placed or stacked<br>If chair/stool is rolled across the room, code *Equip* (see above).
Bump*	*Bump**	Staff/ Visitor/ Extern bump unintentionally into movable objects like garbage bin, chair, trolley, bed, divider, etc.<br>• Do not specify what was bumped.<br>If objects are intentionally moved, then code it in the respective category (see above/below).
Object drops*	*Drop**	Object falls or topples over.<br>• Do not specify what fell
…	...	…
B3. Maintenance		
Laundry/ garbage bins*	*Lid**<br>*Laundry**<br>*Trash**	*Lid* = lid of the laundry/garbage bin falls closed<br>*Laundry* = laundry is thrown in the bin, making rustling sounds<br>*Trash* = garbage is thrown in the bin, making rustling sounds<br>If laundry/garbage bin is rolled across the room, code *Equip* (see above).
Empty laundry/garbage bins	*Empty*	Sounds due to collecting laundry/garbage bags from bins.<br>• If laundry and garbage are not collected in the same way (e.g., plastic bags), code them in two different categories.
Preparing the patient's place	*Prepare*	All noisy events due to the preparation of the area before patient's arrival: bring and connect the equipment, set up the pendant, prepare material, etc.
Cleaning the patient place	*Clean*	All noisy events due to tidying and cleaning area after patient is discharged.

		• This includes cleaning and putting away the material, cleaning the surfaces and floor, emptying laundry/garbage bins.<br>• Do not code separately *Prep*, *Equip*, *Pendant*, *Lid*, *Trash,* and *Laundry* (see above)
Floor cleaning machine	*Floor*	Sounds from industrial floor cleaner (i.e., not just a broom or mop).
…	…	…
B4. Personal items		
Entertainment	*Entertain*	Sounds from entertainment (TV, radio, music, audiobook, etc.)
Patient stuff	*Pat-stuff*	All noisy events due to independent activities of awake Patient: use personal items, sort through bag, etc.
Clothing accessories	*Rattle*<br>*Shoes*	*Rattle* = rattle of clothing, keys, badge, pager, pens, etc. against each other<br>*Shoes* = loud footsteps across the room and/or squeaky shoes
Ringing*	*Pager**<br>*Phone**<br>*Mobile**	*Pager* = pager rings<br>*Phone* = work telephone rings or telephone handset is put back<br>*Mobile* = private mobile telephone of Staff/ Patient/ Visitor rings
Observer's sound*	*O-sound**	All short-lasting sound due to the observer (member of the study team) and their personal equipment: pen clicking/falling, squeaky chair, bump, etc.
…	…	…

Supplementary Table 2. Categories of the occurred sound source analyses. Sound sources are grouped for ease of analysis. Categories can be adapted depending how detailed of an analysis is desired. Note that only the sound sources which have occurred during the 24-hour observation period are mentioned. An asterix (*) indicates the short-lasting sound sources

	**Categories**	**Included sound sources (abbreviation)**
**Human (-human interaction) sounds**	A1. Verbal	
	Staff < 3 people talking (out of ward round)	*S, E, Shout, SPhone, SS, SPh, SE*
	Staff ≥ 3 people talking (out of ward round)	*SSS, SSPh, SSSS, 5S, 6S, 7S,*
	Staff during ward round < 3 people talking	*Round S, Round SS*
	Staff during ward round ≥ 3 people talking	*Round SSS, Round SSSS, Round 5S*
	Patient talking	*SPat, PhPat, SSPat,*
	Visitors talking	*VV, VVPat, SVV, SVVPat, SSVV, SSVVPat*
	A2. Non-verbal	
	Staff sounds	*S-sound, S-cough*
	Patient sounds	*Pat-sound*
**Object (-human interaction) sounds**	B1. Patient care	
	Admission and discharge	*Admission, Discharge*
	Activity of daily living:<br>- Non-mobilization	 *Int. washing, Int. water spray*
	Activity of daily living:<br>- Mobilization	 *Int. mobilizing, Int. physiotherapy*
	Nursing	*Int. bandage*
	Diagnostic:<br>- Standard monitoring<br>- Radiological diagnostic	 *Int. temperature, Int. blood pressure, Int. ECG*<br>*Int. X-ray, Int. sonography*
	Oxygen	*Int. oxygen*
	Suctioning	*Int. suctioning*
	Intubation and extubation	*Int. extubation*
	Water seal (cardiotomy reservoir)	*Int. water seal drainage*
	Renal replace therapy	*Int. RRT*
	Unknown intervention	*Int. unknown*
	B2. General activities	
	Preparation board	*Prep1, Prep2, Water*
	Fee standing equipment	*Equip*
	Pendant	*Pendant*
	Bed-related:<br>- Bed<br>- Changing bedsheets	 *Bed*<br>*Int. changing bedsheet*
	Privacy screens	*Door, Divider*

	Short-lasting activities*	*Bump*, Drop*, Chair**
	B3. Maintenance	
	Continuous maintenance	*Empty, Clean*
	Short-lasting maintenance*	*Lid*, Laundry*, Trash**
	B4. Personal items	
	Clothing accessories	*Rattle, Shoes*
	Ringing*	*Pager*, Phone**
	C. Alarms	
	Monitor alarms	

Supplementary Table 3. Full results per shift, device, and day. Each shift represents an eight-hour recording period. Hallways indicates the device at the nurses' station, Bed – 1 indicates the device above bed 1, Bed - 2 indicates the device above bed 2, and Bed - Neighbor indicates the device above the neighboring bed (to the left of bed 1 in figure 3).

		**L_{Aeq} (dBA)**			**L_{FAmax} (dBA)**		
		Day	Evening	Night	Day	Evening	Night
**Day 1**	Hallway	52.55	51.06	49.00	76.50	85.59	77.47
	Bed – 1	54.45	52.62	49.31	72.10	72.30	67.40
	Bed – 2	53.66	50.97	46.80	70.00	70.70	64.20
	Bed - Neighbor	54.27	53.37	51.04	71.50	75.10	69.00
**Day 8**	Hallway	53.88	52.96	47.80	83.13	75.82	77.49
	Bed – 1	54.01	54.34	47.06	74.50	70.00	69.80
	Bed – 2	53.92	52.97	45.47	72.20	72.90	68.70
	Bed - Neighbor	56.73	55.15	48.09	76.80	69.90	64.40
**Day 15**	Hallway	53.17	52.27	49.60	80.32	75.89	74.43
	Bed – 1	54.45	53.05	51.81	77.50	71.40	70.00
	Bed – 2	52.40	52.29	50.86	72.60	71.10	72.70
	Bed - Neighbor	56.20	54.35	51.48	83.90	73.10	70.30
**Day 22**	Hallway	53.48	53.18	49.48	77.18	77.03	75.94
	Bed – 1	53.67	51.20	46.81	67.80	69.40	65.90
	Bed – 2	51.73	50.71	47.68	68.00	68.50	70.30
	Bed - Neighbor	54.95	52.41	47.55	71.60	72.50	65.60

Supplementary Table 4. Full overall, day, evening, and night shift, results for all categories at bed 1 and bed 2. Where there is no minute or percent value given, the number represents the number of occurrences. N/A means there was no occurrence of the category at that bed.

	Description	Bed 1				Bed 2			
		Overall	Day	Evening	Night	Overall	Day	Evening	Night
Human (-Human) Sounds	Staff < 3 people talking (out of ward round)	299 min 23.73%	93 min 21.88%	148 min 35.66%	58 min 13.81%	284 min 22.54%	174 min 40.94%	102 min 24.58%	8 min 1.90%
	Patient talking	141 min 11.19%	0 min 0.00%	47 min 11.33%	48 min 11.43%	116 min 9.21%	53 min 12.47%	47 min 11.33%	16 min 3.81%
	Staff ≥ 3 people talking (out of ward round)	67 min 5.32%	28 min 6.59%	39 min 9.40%	0 min 0.00%	91 min 7.22%	79 min 18.59%	12 min 2.89%	0 min 0.00%
	Patient sounds	25 min 1.98%	0 min 0.00%	6 min 1.45%	19 min 4.52%	55 min 4.37%	27 min 6.35%	9 min 2.17%	19 min 4.52%
	Staff sounds	4 min 0.32%	0 min 0.00%	4 min 0.96%	0 min 0.00%	17 min 1.35%	1 min 0.24%	15 min 3.61%	1 min 0.24%
	Staff during ward round ≥ 3 people talking	7 min 0.56%	5 min 1.18%	2 min 0.48%	0 min 0.00%	7 min 0.56%	5 min 1.18%	2 min 0.48%	0 min 0.00%
	Staff during ward round < 3 people talking	3 min 0.24%	0 min 0.00%	3 min 0.72%	0 min 0.00%	4 min 0.32%	1 min 0.24%	3 min 0.72%	0 min 0.00%
Object (-Human Interaction) Sounds	Oxygen	279 min 22.14%	0 min 0.00%	149 min 25.90%	130 min 30.95%	272 min 21.59%	0 min 0.00%	142 min 34.22%	130 min 30.95%
	Pendant	193 min 15.32%	63 min 14.82%	81 min 19.52%	49 min 11.67%	212 min 16.83%	94 min 22.12%	102 min 24.58%	16 min 3.81%
	Preparation board	290 min 23.02%	91 min 21.41%	119 min 28.67%	80 min 19.05%	101 min 8.02%	62 min 14.59%	34 min 8.91%	5 min 1.19%
	Monitor Alarms	245 min 17.6%	52 min 10.8%	59 min 12.3%	143 min 29.8%	177 min 12.3%	45 min 9.4%	72 min 15.0%	59 min 12.3%
	Clothing accessories	126 min 10.00%	57 min 13.41%	33 min 7.95%	36 min 8.57%	126 min 10.00%	74 min 17.41%	40 min 9.64%	12 min 2.86%
	Free standing equipment	89 min 7.06%	36 min 8.47%	37 min 8.92%	16 min 3.81%	132 min 10.48%	69 min 16.24%	58 min 13.98%	5 min 1.19%

	**Diagnostic**	40 min<br>3.17%	9 min<br>2.12%	21 min<br>5.06%	10 min<br>2.38%	128 min<br>10.16%	114 min<br>26.82%	14 min<br>3.37%	0 min<br>0.00%
	**Bed-related**	41 min<br>3.25%	11 min<br>2.59%	23 min<br>5.54%	7 min<br>1.67%	54 min<br>4.29%	38 min<br>8.94%	16 min<br>3.86%	0 min<br>0.00%
	**Water seal (cardiotomy reservoir)**	N/A	N/A	N/A	N/A	80 min<br>6.35%	80 min<br>18.82%	0 min<br>0.00%	0 min<br>0.00%
	**Renal replace therapy**	N/A	N/A	N/A	N/A	35 min<br>2.78%	34 min<br>8.00%	1 min<br>0.24%	0 min<br>0.00%
	**Suctioning**	25 min<br>1.98%	19 min<br>4.47%	6 min<br>1.45%	0 min<br>0.00%	9 min<br>0.71%	3 min<br>0.72%	6 min<br>1.45%	0 min<br>0.00%
	**Unknown intervention**	18 min<br>1.43%	0 min<br>0.00%	18 min<br>4.34%	0 min<br>0.00%	2 min<br>0.48%	1 min<br>0.24%	1 min<br>0.24%	0 min<br>0.00%
	**Continuous maintenance**	6 min<br>0.48%	6 min<br>1.41%	0 min<br>0.00%	0 min<br>0.00%	15 min<br>1.19%	14 min<br>3.29%	1 min<br>0.24%	0 min<br>0.00%
	**Admission and discharge**	N/A	N/A	N/A	N/A	19 min<br>1.51%	19 min<br>4.47%	0 min<br>0.00%	0 min<br>0.00%
	**Activity of daily living: non-mobilization**	7 min<br>0.56%	3 min<br>0.71%	1 min<br>0.24%	3 min<br>0.71%	11 min<br>0.87%	8 min<br>1.88%	3 min<br>0.72%	0 min<br>0.00%
	**Activity of daily living: mobilization**	8 min<br>0.63%	6 min<br>1.41%	0 min<br>0.00%	2 min<br>0.48%	2 min<br>0.48%	0 min<br>0.00%	2 min<br>0.48%	0 min<br>0.00%
	**Privacy screens**	11 min<br>0.87%	2 min<br>0.47%	7 min<br>1.69%	2 min<br>0.48%	1 min<br>0.08%	0 min<br>0.00%	1 min<br>0.24%	0 min<br>0.00%
	**Intubation and extubation**	3 min<br>0.24%	0 min<br>0.00%	3 min<br>0.72%	0 min<br>0.00%	2 min<br>0.48%	0 min<br>0.00%	2 min<br>0.48%	0 min<br>0.00%
	**Nursing**	2 min<br>0.16%	0 min<br>0.00%	2 min<br>0.48%	0 min<br>0.00%	5 min<br>0.40%	4 min<br>0.94%	1 min<br>0.24%	0 min<br>0.00%
	**Short-lasting activities**	100x	28x	50x	22x	104x	48x	47x	9x
	**Short-lasting maintenance**	99x	50x	42x	7x	104x	38x	38x	1x
	**Ringing**	13x	5x	6x	2x	25x	16x	9x	0x
